# The Arizona Sexual Experiences Scale-the Thai Translation (ASEX-Thai): Reliability and validity in Thai patients with mental disorders

**DOI:** 10.12688/f1000research.111051.2

**Published:** 2022-10-04

**Authors:** Natthaphon Charoenmakpol, Mayteewat Chiddaycha, Sorawit Wainipitapong

**Affiliations:** 1 Department of Psychiatry, Faculty of Medicine, Chulalongkorn University and King Chulalongkorn Memorial Hospital, the Thai Red Cross Society, Bangkok, 10330, Thailand; 2 Center of Excellence in Transgender Health (CETH), Faculty of Medicine, Chulalongkorn University, Bangkok, 10330, Thailand

**Keywords:** Arizona Sexual Experiences Scale, ASEX, sexual dysfunction, Thai, mental disorder, psychiatric disorder

## Abstract

**Background: **Sexual dysfunction is common among patients with mental disorders but receives less clinical attention, especially in Thailand and other Asian countries. The Arizona Sexual Experiences Scale-the Thai Translation (ASEX-Thai), a self-rated, brief, questionnaire is a potential tool for screening for sexual dysfunction in this population. Our study aimed to assess the reliability and validity of ASEX-Thai in Thai patients with mental disorders.

**Methods: **We enrolled 202 patients from an outpatient psychiatric department at a tertiary hospital in Bangkok, Thailand. Demographic data, clinical data, and diagnosis of sexual dysfunction were assessed. ASEX-Thai was done, and we analyzed the test’s psychometric properties.

**Results: **Most participants were diagnosed with major depressive disorder (48%). There was a positive correlation between the ASEX-Thai and sexual dysfunction diagnosis (r = 0.402, p < 0.001). The KMO coefficient was 0.77 and Barlett’s sphericity test was significant (χ ^2^ = 409.76, p<0.001). A score of ≥ 17 points of the ASEX-Thai was the most suitable for sexual dysfunction screening (sensitivity 77.23 %, and specificity 58.42 %). For reliability, the Cronbach’s alpha coefficient (0.831) showed good internal consistency.

**Conclusions: **The ASEX-Thai is a valid and reliable self-rated questionnaire for screening for sexual dysfunction among Thai patients with mental disorders. The test could help clinicians to evaluate this undetected condition and deliver proper interventions.

## Introduction

The World Health Organization (WHO) has emphasized the importance of mental health with its famous statement ‘without mental health there can be no true physical health’. ^1^ There has been stronger evidence of the bidirectional linkage between mental and physical health. Mental disorders are significantly associated with higher hospitalizations, longer length of stay, and increased healthcare costs due to physical diseases. ^2^ Additionally, certain physical diseases may precede the onset of mental disorders and vice versa. ^3^ The adverse effects of medications for these two conditions also portray the same interaction. Apart from other physical causes, sexual problems are also commonly found among those receiving psychotropic medications. ^4^ Sexual dysfunction (SD) is not only a frequent adverse effect but could also be a manifestation of some psychiatric disorders. Moreover, SD itself could be comorbid with other non-sexual mental disorders. ^5^ Sexual dissatisfaction markedly affects the quality of life and mental health, ^6^ therefore it may be reasonable to derive from the WHO’s statement that ‘there also can be no true mental health without sexual health’.

SD has been underrecognized in some Asian countries according to different cultures and attitudes toward sexuality. ^7^ The prevalence of SD is mainly studied in some specific physical morbidities and tends to receive less academic attention in psychiatric populations. Some studies reported a great number of SD (60.7-82.7%) among those with schizophrenia, ^8^^,^^9^ but related findings are still lacking in Asians with other mental disorders. A validated, especially a self-rated, SD measurement would be beneficial in terms of research and clinical application in the Asian culture where sexual issues are considered underreported and untreated. ^7^

The Arizona Sexual Experiences Scale (ASEX) is a 5-item self-rated, brief questionnaire with a 6-point Likert scale assessing SD components including desire, arousal, penile erection or vaginal lubrication, ability to achieve orgasm and orgasmic satisfaction. ^10^ The ASEX can be used regardless of sexual orientation and individual or partnered sexual activity. However, some items are not suitable for specific populations, such as transgender people, especially after the transition. The score is positively correlated with the severity of SD, and it can be implemented in various clinical settings with good reliability. ^11^ The cutoff score is varied due to the methodology and study populations in each study. ^10^^–^^14^ Validation studies have been done in some psychiatric and physical diseases. The ASEX is available in 43 languages and a few non-English versions, including Thai, of ASEX have been examined for their psychometric properties. However, only the Arabic and French versions of the ASEX have been validated in patients with psychiatric disorders. ^12^^,^^15^ Psychometric properties from these two versions are highly acceptable. However, the application of the Arabic and French versions is limited since the study was done in patients with schizophrenia and depression, respectively. Only the original ASEX was assessed for its reliability and validity in those with several mental disorders, so the generalizability of other translated versions in this certain population is still lacking.

The ASEX- Thai Translation (ASEX-Thai) was assessed for its reliability and validity among patients with Parkinson’s disease. ^14^ The study included a forward/backward translation, and cross-cultural modifications for assuring content validity. The Cronbach’s alpha of all items with values of 0.948 at baseline and 0.962 at 2-month follow-up confirmed the reliability of the ASEX-Thai. Those with scores over fifteen would be considered positive for SD with 96.2% and 92.9% of sensitivity and specificity, respectively. However, the generalizability of the cutoff point is limited regarding the study population. Patients with Parkinson’s disease also contain ample SD risk factors, including age, motor deterioration, psychiatric comorbidities, and neuropsychiatric medications. A validation of ASEX-Thai in disparate populations would be profitable in clinical practices, especially among patients whose risks for SD are critically high, including psychiatric patients. Hence, our study aimed to examine the validity and reliability of ASEX-Thai in Thai patients with mental disorders.

## Methods

We enrolled participants aged at least 18 years old, from the psychiatric outpatient department at King Chulalongkorn Memorial Hospital in Bangkok, Thailand. All participants received a steady dosage of medications for at least one month and were diagnosed with mental disorders including schizophrenia, major depressive disorder, bipolar disorder, obsessive-compulsive disorder, or anxiety disorders. Those who were unable to communicate properly and had unstable medical or psychiatric conditions were excluded. Sample size calculation was done based on sensitivity, specificity and the results from the previous study. ^10^^,^^16^ With SD prevalence of 31%, 82% and 90% of sensitivity and specificity, respectively, we used Buderer’s method for sample size calculation, which required a minimum total sample of 202 patients. Kaiser-Meyer-Olkin (KMO) and Barlett’s sphericity test confirmed that the sample size was adequate for the factor analysis. Written informed consent for participation and publication of the participants’ details was obtained from all participants was obtained, and the study was approved by the Institutional Review Board of the Faculty of Medicine, Chulalongkorn University, Bangkok, Thailand (IRB No. 430/2020).

Demographic data, including sex, age, body mass index, psychotropic medications, and medical history were collected. The severity of their mental disorder was assessed using the Brief Psychiatric Rating Scale (BPRS). We used the second version of BPRS, which consists of 18 items measuring multidimensions of psychiatric symptoms, and categorized into two grades of severity with a cutoff score of 36. ^17^

The ASEX-Thai was used under the permission of the original researcher. Diagnosis of SD based on the Diagnostic and Statistical Manual of Mental disorder, Fifth edition (DSM-5) was evaluated for establishing criterion validity for SD of ASEX-Thai. After completing the questionnaire, all participants were assessed their SD diagnosis by the psychiatric interview, while the ASEX-Thai result remained concealed. The interview was conducted by one psychiatrist to limit inter-rater bias. According to DSM-5, SD diagnoses include male hypoactive sexual desire disorder, erectile disorder, premature (early) ejaculation, delayed ejaculation, female sexual interest/arousal disorder, female orgasmic disorder, genito-pelvic pain/penetration disorder, and substance/medication-induced SD. Either medical conditions or non-sexual mental disorders should be excluded, and SD symptoms must cause significant functional impairment or individual distress. Test-retest reliability was not done since SD is impacted by multiple factors and may suddenly occur within a short period. ^18^ Content validity was proved in the previous study. ^14^

Descriptive statistics were used to report the demographic data of all participants. Categorical variables were presented as counts and percentage, and continuous variables were shown as appropriate. In order to determine the criterion validity, we used Pearson’s correlation to assess the correlation between SD diagnosis based on ASEX-Thai and DSM-5. Exploratory factor analysis with principal-components method and varimax rotation was conducted to test the construct validity. Internal consistency was tested by the Cronbach’s alpha coefficient. Omega’s coefficient was also calculated. Receiver operating characteristic (ROC) analysis was used to determine the area under curve (AUC). The sensitivity, specificity, and Youden J index were calculated for every cut-off point of the ASEX-Thai to find the optimal cut-off for sexual dysfunction screening. A *p* value of < 0.05 was considered statistically significant. STATA-IC Version 16.1 was used for analysis.

## Results

In total, 202 participants were recruited throughout our study period. Male participants were slightly greater in number (54.9%) and the median age was 28 years old. Most participants had normal body weight (Body mass index 23.8 kg/m ^2^) and hypertension was the most frequent medical comorbidity (2.9%). As for psychiatric disorders, major depressive disorder remained the highest in number (48.0%) followed by anxiety disorder (18.3%) and schizophrenia (15.3%). According to the BPRS, the majority appeared to have mild symptoms (92.1%). The demographic data and psychiatric history are displayed in Table 1.

**Table 1.** Demographic data.

Participants (N=202)	N (%)
Male	111 (55)
Age (median [Interquartile range])	28.0 [23.0, 34.2]
Body mass index (mean ± SD)	24.3 ± 4.8
Marital status	
- Single	164 (81.2)
- Married/coupled	32 (15.8)
- Separated/divorced/widowed	6 (3.0)
Participants with physical comorbidities	9 (9.9)
Psychiatric diagnosis	
- Major depressive disorder	97 (48.0)
- Anxiety disorder	37 (18.3)
- Schizophrenia	31 (15.3)
- Bipolar disorder	21 (10.9)
- Obsessive-compulsive disorder	16 (7.9)
Psychotropic medication use	
- Antipsychotic drug	65 (32.2)
- Antidepressant	164 (81.2)
- Mood stabilizer/anticonvulsant	26 (12.9)
- Sedative/Hypnotic drug	95 (47.0)
- Anticholinergic agent (Trihexyphenidyl)	22 (10.9)
Brief Psychiatric Rating Scale	
- Mild symptoms	186 (92.1)
- Severe symptoms	16 (7.9)
- Mean ± SD	26.6 ± 7.3

Regarding the DSM-5 criteria, SD was found in 101 participants (50.0%). Pearson’s correlation portrayed a weak correlation between the ASEX-Thai positive for SD and clinical diagnosis according to the DSM ( *r* = 0.402, *p* < 0.001).

The KMO coefficient was 0.77, which was above 0.50, and the result of Barlett’s sphericity test was found to be statistically significant (χ ^2^ = 409.76; *p* < 0.001). Therefore, the sample size was sufficient for the analysis. Exploratory factor analysis showed one factor that explained 60.2% of the total variance. Factor loadings ranged from 0.72 to 0.86.

Area under the curve (AUC) of the ROC analysis showed the ability to distinguish between those with and without SD (0.75 ± 0.03, *p* < 0.001). Among patients with mental disorders, we found that a cutoff score of ≥ 17 points of the ASEX-Thai was the most suitable for SD screening (sensitivity 77.23 %, specificity 58.42%, Youden J index 0.40). According to the manual of the ASEX, two additional proposed criteria, which indicated positive SD screening, were calculated with the ROC analysis. Evaluating participants who (1) had three or more items with an individual score of 4, or (2) any individual score of either 5 or 6, we found that the AUC for (1) and (2) was 0.68 (95% CI 0.61-0.74) and 0.75 (95% CI 0.69-0.81) respectively.

For the reliability, the Omega coefficient was 0.83. The Cronbach’s alpha coefficient was greater than 0.7 which indicated good internal consistency.

## Discussion and conclusion

SD among patients with mental disorders is common but undetected. ^19^ Sexuality assessment is considered uncomfortable for healthcare providers because of stigmatization and limited opportunities for practice ^20^ especially in Asian cultures, which are comparatively more conservative. ^21^ This important problem can potentially be avoided by the use of a self-rated questionnaire.

The ASEX-Thai, a brief and self-rated measurement, showed good reliability and validity for SD assessment among patients with Parkinson’s disease and mental disorders ( Table 2).

**Table 2.** The comparison between two ASEX-Thai studies and the original ASEX.

	Our study	Jitkritsadakul et al. ^14^	McGahuey et al. ^10^
Year of study	2020-2021	2012	2000
Study population	Mental disorders	Parkinson’s disease	Mental disorders
Setting	General Psychiatry outpatient department	Movement disorders outpatient clinic	-
Number of participants	202	40	58
Validity			
- Cutoff score	17	16	19
- Area under the curves (AUCs)	0.75 ± 0.03	0.927 ± 0.05	0.929 ± 0.03
- Sensitivity	77.23%	96.2%	82.0%
- Specificity	58.42%	92.9%	90.0%
- Pearson’s correlation	DSM-5 *r* 0.402, *p* < 0.001	DSM-IV-TR *r* 0.601, *p* < 0.001	BISF ^a^ *r* 0.801, *p* < 0.01
Reliability			
- Cronbach’s alpha coefficient	0.831	0.948	0.9055

^a^ *BISF* Brief Index of Sexual Functioning.

With a cutoff score of 17, the ASEX-Thai revealed constraints regarding its inferior psychometric properties. However, the purpose of the questionnaire was for SD screening, therefore lower specificity is considered acceptable, as one previous study had selected its cutoff for screening at 11 (sensitivity 100%, specificity 52%). ^13^ Proper psychiatric assessment should be further evaluated for the diagnosis of SD. Adding two more criteria for a positive ASEX-Thai (those who had three or more items with individual score of 4 or had any 5 or 6 for at least one item) could improve the questionnaire’s property (sensitivity 82.2% specificity 55.4%).

The lower specificity found in our study could be explained by the use of DSM-5 for validation. The diagnosis of SD according to DSM-5 consists of one essential criterion regarding the individual’s clinically significant distress; meanwhile, the ASEX-Thai defines a positive screening by persistence of other symptoms alone, which may not fulfill the DSM-5 SD diagnostic criteria. This linkage was confirmed by a weak correlation from Pearson’s correlation. Also, the repression of sexual satisfaction and sex guilt among the Asian population is common, and lower desire is reported. ^22^^,^^23^ Consequently, the distress of each individual may be obscured, and the diagnosis cannot be fulfilled. Our participants were also mostly young and single; thus, distress or impairment in interpersonal function might be absent. Supplemental items underpinning participants’ distress or impairment could further enhance the psychometric property of ASEX-Thai.

All participants were assessed by one clinician, so interrater bias of DSM-5 diagnosis was subtle. Our study reported poorer Cronbach’s alpha when compared with the previous ASEX-Thai and original ASEX studies. Since the ASEX-Thai measured every aspect of sexual function according to the human sexual response cycle, the extent of the participants’ sexual experiences could be a limitation, especially for those who have never engaged in partnered sexual activities. Their scores from these associated items might then be lower. We collected a large sample size that included psychiatric patients with various diagnoses, age, age of onset, and severity. These variables might impact the patient’s self-disclosure and cause self-reporting bias, which can interfere with the reliability of the ASEX-Thai, which is a self-rated questionnaire. ^24^

Our participants were diagnosed with certain mental disorders and were prescribed psychotropic medications from their attending psychiatrists. Multiple studies suggested that psychiatric illness and its treatment are related to the development of SD. ^25^^–^^27^ However, receiving substances or medications able to induce SD does not exclude its diagnosis. ^28^ Therefore, the nature of our participants does not confound the validity of the ASEX-Thai but increases its utility because of the high prevalence of SD among this population. ^29^

Compared to prior versions of ASEX studies in other languages, our study covered the greatest sample size and diversity in psychiatric diagnoses. The severity of psychiatric symptoms of all participants were assessed with the same, standardized, questionnaire by a sole investigator and a rater bias was minimized.

Some limitations should be mentioned. The number of each psychiatric diagnosis was not distributed equally and could limit the generalizability to the whole psychiatric outpatient department and patients with mild symptoms. The majority of our participants were diagnosed with major depressive disorder, similar to the original version of the ASEX in psychiatric patients. ^10^ However, the ASEX-Thai can be implemented as a screening tool and can facilitate psychiatrists to further evaluate SD and make a diagnosis using the psychiatric interview and gold-standard DSM-5 criteria. Some items of the ASEX-Thai cannot assess SD in specific populations, such as transitioned transgender people. Limitation and complications of gender-affirming therapy include lubrication in neovagina or erection in neophallus constructed by phalloplasty *.* ^30^^,^^31^ Future studies focusing on clinical applications of the ASEX-Thai are needed to evaluate SD, both primary and medication-induced in etiology, among patients with mental disorders and gender diversity.

Patients with mental disorder suffer from their mental health and underrecognized sexual problems, which may partly be caused by treatment complications. This undetected and untreated condition should be emphasized congruently with the cultural context and comfort of both patients and clinicians. The ASEX-Thai is a brief, self-rated, valid, and reliable questionnaire for SD screening among Thai patients with mental disorders.

## Data availability

The data that support the findings of this study are not publicly available due to the containing information that is highly sensitive (participants’ mental and sexual health). However, the data can be available on request via email to the corresponding author. The access to the dataset is granted only for the purpose of reviewing and academic reasons.
