# Supplementary material for: The Arizona Sexual Experiences Scale-the Thai Translation (ASEX-Thai): Reliability and validity in Thai patients with mental disorders
Source: F1000Res. 2022 Oct 4;11:503. Originally published 2022 May 9. [Version 2] doi: 10.12688/f1000research.111051.2 (PMC9525992; doi:10.12688/f1000research.111051.2)
Supplement: Peer review history

# Peer review: Reviewer response for version 2

**DOI:** 10.5256/f1000research.139311.r152385

**Published:** 2022-10-05

**Authors:** Gonzalo R Quintana

**Affiliations:** 1 Quintana, Departamento de Psicología y Filosofía, Facultad de Ciencias Sociales y Jurídicas, Universidad de Tarapacá, Arica, Chile

The authors responded in a satisfactory way to every suggestion / concern, except for one. The IIEF contains in many items the answer 0 (i.e., No sexual activity, Did not attempt intercourse, No attempts, No sexual stimulation or intercourse), from question 1 until 9. From these responses the authors are able to re-evaluate to either take every subject out, create a different group, or establish a criterion as to who was really not sexually active. I believe these analyses are feasible.

Is the work clearly and accurately presented and does it cite the current literature?

Yes

If applicable, is the statistical analysis and its interpretation appropriate?

Yes

Are all the source data underlying the results available to ensure full reproducibility?

Yes

Is the study design appropriate and is the work technically sound?

Yes

Are the conclusions drawn adequately supported by the results?

Yes

Are sufficient details of methods and analysis provided to allow replication by others?

Yes

Reviewer Expertise:

NA

I confirm that I have read this submission and believe that I have an appropriate level of expertise to confirm that it is of an acceptable scientific standard, however I have significant reservations, as outlined above.

# Peer review: Reviewer response for version 2

**DOI:** 10.5256/f1000research.139311.r152383

**Published:** 2022-10-05

**Authors:** Chotiman Chinvararak

**Affiliations:** 1 Department of Psychiatry, Faculty of Medicine Vajira Hospital, Navamindradhiraj University, Bangkok, Thailand

Thank you very much for inviting me to review this article.

 The authors have addressed all issues of my comment.

Is the work clearly and accurately presented and does it cite the current literature?

Yes

If applicable, is the statistical analysis and its interpretation appropriate?

I cannot comment. A qualified statistician is required.

Are all the source data underlying the results available to ensure full reproducibility?

Yes

Is the study design appropriate and is the work technically sound?

Yes

Are the conclusions drawn adequately supported by the results?

Yes

Are sufficient details of methods and analysis provided to allow replication by others?

Yes

Reviewer Expertise:

Psychiatry

I confirm that I have read this submission and believe that I have an appropriate level of expertise to confirm that it is of an acceptable scientific standard.

# Peer review: Reviewer response for version 2

**DOI:** 10.5256/f1000research.139311.r152384

**Published:** 2022-10-04

**Authors:** Pakawat Wiwattanaworaset

**Affiliations:** 1 Department of Psychiatry, Faculty of Medicine, Prince of Songkla University, Hat Yai, Thailand

Thank you for this revision. I have no further comments.

Is the work clearly and accurately presented and does it cite the current literature?

Yes

If applicable, is the statistical analysis and its interpretation appropriate?

I cannot comment. A qualified statistician is required.

Are all the source data underlying the results available to ensure full reproducibility?

Yes

Is the study design appropriate and is the work technically sound?

Yes

Are the conclusions drawn adequately supported by the results?

Yes

Are sufficient details of methods and analysis provided to allow replication by others?

Yes

Reviewer Expertise:

Psychiatry

I confirm that I have read this submission and believe that I have an appropriate level of expertise to confirm that it is of an acceptable scientific standard.

# Peer review: Reviewer response for version 1

**DOI:** 10.5256/f1000research.122724.r151715

**Published:** 2022-09-30

**Authors:** Gonzalo R Quintana

**Affiliations:** 1 Quintana, Departamento de Psicología y Filosofía, Facultad de Ciencias Sociales y Jurídicas, Universidad de Tarapacá, Arica, Chile

**Overall**

 Charoenmakpol et al. Provided the psychometric evaluation (reliability and validity) for the Arizona Sexual Experiences Scale in Thai for patients with mental disorders. They showed that the instrument has adequate sensitivity and reliability, yet not good specificity, which relates to the low correlation with the criterion. The authors discuss why these results do not replicate previously reported better specificity.

**Specific comments**

**Abstract **

- It is well formatted. I would include information regarding the model fit and its validity assessed.

**Introduction **

- I believe the last sentence of the 1st paragraph may benefit by changing “add” to “expand” or “derive from the WHO...”
- A citation seems necessary for the last sentence of the 2nd paragraph. Otherwise, reconsider "forbidden”
- In the last sentence of the 3th paragraph, it says “Only the original ASEX was assessed its reliability...” This needs rewording. It appears that is missing the “for” before “its”.

**Methods **

- The study does not include a description of a Ethical Evaluation. This is of great concern, especially due to the sample characteristics
- Some readers would benefit from an explanation of the IQR acronym
- There is no reason not to include the Omega’s calculation for reliability

**Discussion**

- The authors do a good job discussing the findings and limitations.
- Whereas the scale may not instruct researchers to exclude not-sexually active people, to include these people in the analysis using other sexual function scales has shown to be problematic (e.g., IIEF). If the authors can redo their analysis excluding these individuals, it may be a hypothesis worth considering. If indeed the authors have this information, they must include it, and assess if it may partially explain the results.

Is the work clearly and accurately presented and does it cite the current literature?

Yes

If applicable, is the statistical analysis and its interpretation appropriate?

Yes

Are all the source data underlying the results available to ensure full reproducibility?

Yes

Is the study design appropriate and is the work technically sound?

Yes

Are the conclusions drawn adequately supported by the results?

Yes

Are sufficient details of methods and analysis provided to allow replication by others?

Yes

Reviewer Expertise:

Sex, sexual behavior and function

I confirm that I have read this submission and believe that I have an appropriate level of expertise to confirm that it is of an acceptable scientific standard, however I have significant reservations, as outlined above.

# Response

**Published:** 2022-10-01

**Authors:** Sorawit Wainipitapong

**Affiliations:** Chulalongkorn University, Thailand

Thank you for your immensely helpful suggestions. Please find my point-by-point responses below:

**Abstract**

 1) It is well formatted. I would include information regarding the model fit and its validity assessed.

 Response –Thank you for your suggestion. Information was added into the abstract.

**Introduction**

 1) I believe the last sentence of the 1st paragraph may benefit by changing “add” to “expand” or “derive from the WHO...”

 Response – Thank you. We have now changed to “derive from” as your suggestion.

 2) A citation seems necessary for the last sentence of the 2nd paragraph. Otherwise, reconsider "forbidden”

 Response – Thank you for this crucial point. We have changed to “underreported and untreated” and the citation has been added.

 3) In the last sentence of the 3th paragraph, it says “Only the original ASEX was assessed its reliability...” This needs rewording. It appears that is missing the “for” before “its”.

 Response – Thank you. “For” has now been added.

**Methods**

 1) The study does not include a description of a Ethical Evaluation. This is of great concern, especially due to the sample characteristics

 Response – Ethical issue is also our major concern. Our study received the approval from our institution’s IRB. We have mentioned about this in the last sentence of the first paragraph of the method section.

 2) Some readers would benefit from an explanation of the IQR acronym

 Response – Thank you very much. The acronym has now been clarified.

 3) There is no reason not to include the Omega’s calculation for reliability

 Response – Thank you for your suggestion. We have calculated the Omega’s reliability. The result of Omega’s reliability was 0.83 and we have added it into the method and result section.

**Discussion**

 1) Whereas the scale may not instruct researchers to exclude not-sexually active people, to include these people in the analysis using other sexual function scales has shown to be problematic (e.g., IIEF). If the authors can redo their analysis excluding these individuals, it may be a hypothesis worth considering. If indeed the authors have this information, they must include it, and assess if it may partially explain the results.

 Response - Thank you for the suggestion. We totally agree that our result would be much more interesting regarding additional analysis. Unfortunately, active sexual activity was not collected in our dataset. However, we will recruit your suggested variables in our further studies.

# Peer review: Reviewer response for version 1

**DOI:** 10.5256/f1000research.122724.r140559

**Published:** 2022-09-29

**Authors:** Pakawat Wiwattanaworaset

**Affiliations:** 1 Department of Psychiatry, Faculty of Medicine, Prince of Songkla University, Hat Yai, Thailand

This article is interesting and will be useful for screening people with sexual dysfunction in Thailand. However, I have some comments that may improve the scientific value of this article.

1. Most participants were diagnosed with major depressive disorder, in other words, most of them received antidepressants which could cause sexual dysfunction. Did it confound the result of this study?  
2. Most participants had mild BPRS. Do you think that the result of this study can apply in all levels of psychiatric patients (mild to severe symptoms)?  Please give further discussion.

Is the work clearly and accurately presented and does it cite the current literature?

Yes

If applicable, is the statistical analysis and its interpretation appropriate?

I cannot comment. A qualified statistician is required.

Are all the source data underlying the results available to ensure full reproducibility?

Yes

Is the study design appropriate and is the work technically sound?

Yes

Are the conclusions drawn adequately supported by the results?

Yes

Are sufficient details of methods and analysis provided to allow replication by others?

Yes

Reviewer Expertise:

Psychiatry

I confirm that I have read this submission and believe that I have an appropriate level of expertise to confirm that it is of an acceptable scientific standard.

# Response

**Published:** 2022-10-01

**Authors:** Sorawit Wainipitapong

**Affiliations:** Chulalongkorn University, Thailand

We appreciate your advice on our manuscript. Hereby, please find our response to your queries.

 1) Most participants were diagnosed with major depressive disorder, in other words, most of them received antidepressants which could cause sexual dysfunction. Did it confound the result of this study?

 Response – Thank you for this interesting question. As your opinions, those receiving antidepressants might have poorer sexual function and SD could be diagnosed. According to DSM-5, medication-induced SD was included in our study. We believe that our psychometric properties would not be confounded from this nature. We have mentioned about this point in the sixth paragraph of the discussion section.

 2) Most participants had mild BPRS. Do you think that the result of this study can apply in all levels of psychiatric patients (mild to severe symptoms)?  Please give further discussion.

 Response – Thank you for this point. Generalizability of the tool was limited to those with mild symptoms. We have now clarified about this in the limitation.

# Peer review: Reviewer response for version 1

**DOI:** 10.5256/f1000research.122724.r144417

**Published:** 2022-08-25

**Authors:** Chotiman Chinvararak

**Affiliations:** 1 Department of Psychiatry, Faculty of Medicine Vajira Hospital, Navamindradhiraj University, Bangkok, Thailand

Thank you very much for inviting me to be a reviewer of this article.

 This article is the first study aimed to validate ASEX-Thai questionnaire in Thai patients with mental disorders. 

 The writing style was done with a standard academic English.

 The authors demonstrated an appropriate rationale, study design and statistical analysis.

 I have few comments that may improve the scientific value of this article.

 1) According to “The KMO coefficient was 0.77, which was above 0.50, and the result of Barlett’s sphericity test was found to be statistically significant (χ ^2^ = 409.76; *p* = 0.000)” please change the p-value to p<0.001

 2) please mention and explain more detail about DSM-5 diagnostic interview for sexual dysfunction in the methods

 (as the authors have discussed already in the discussion part)

 3) please discuss more on the comparison between this study and the study of Jitkritsadakul et al. And the reason why there is a difference in the sensitivity and specificity.

 I think this article has a sufficient value for Indexing.

Is the work clearly and accurately presented and does it cite the current literature?

Yes

If applicable, is the statistical analysis and its interpretation appropriate?

I cannot comment. A qualified statistician is required.

Are all the source data underlying the results available to ensure full reproducibility?

Yes

Is the study design appropriate and is the work technically sound?

Yes

Are the conclusions drawn adequately supported by the results?

Yes

Are sufficient details of methods and analysis provided to allow replication by others?

Yes

Reviewer Expertise:

Psychiatry

I confirm that I have read this submission and believe that I have an appropriate level of expertise to confirm that it is of an acceptable scientific standard, however I have significant reservations, as outlined above.

# Response

**Published:** 2022-10-01

**Authors:** Sorawit Wainipitapong

**Affiliations:** Chulalongkorn University, Thailand

Thank you for your kind and helpful comments. Please find my point-by-point responses below:

 1) According to “The KMO coefficient was 0.77, which was above 0.50, and the result of Barlett’s sphericity test was found to be statistically significant (χ ^2^ = 409.76; *p* = 0.000)” please change the p-value to p<0.001 

 Response – Thank you. We have changed as your suggestion in our revised version.

 2) please mention and explain more detail about DSM-5 diagnostic interview for sexual dysfunction in the methods (as the authors have discussed already in the discussion part)

 Response – Thank you for this point. Additional details about DSM-5’s sexual dysfunction and our clinical interview have been stated in the method section.

 3) please discuss more on the comparison between this study and the study of Jitkritsadakul et al. And the reason why there is a difference in the sensitivity and specificity.

 Response – The psychometric properties from previous ASEX-Thai study were studied among patients with Parkinson’s disease whose clinical and demographical profiles were totally different. Our participants were younger and most of them were single. SD then could not be diagnosed for absence of functional/interpersonal impairment, and this explained a difference of our sensitivity and specificity, compared to previous study. We have mentioned this in Table 2 and the fourth paragraph of the discussion section.
